# Short-Range Mobility and the Evolution of Cooperation: An Experimental Study

**DOI:** 10.1038/srep10282

**Published:** 2015-05-20

**Authors:** Alberto Antonioni, Marco Tomassini, Angel Sánchez

**Affiliations:** 1 Faculty of Business and Economics, University of Lausanne, Switzerland; 2 Grupo Interdisciplinar de Sistemas Complejos (GISC), Departamento de Matemáticas, Universidad Carlos III de Madrid, Leganés, Spain; 3 Instituto de Biocomputación y Física de Sistemas Complejos (BIFI), Universidad de Zaragoza, Spain

## Abstract

A pressing issue in biology and social sciences is to explain how cooperation emerges in a population of self-interested individuals. Theoretical models suggest that one such explanation may involve the possibility of changing one’s neighborhood by removing and creating connections to others, but this hypothesis has problems when random motion is considered and lacks experimental support. To address this, we have carried out experiments on diluted grids with human subjects playing a Prisoner’s Dilemma. In contrast to previous results on purposeful rewiring in relational networks, we have found no noticeable effect of mobility in space on the level of cooperation. Clusters of cooperators form momentarily but in a few rounds they dissolve as cooperators at the boundaries stop tolerating being cheated upon. Our results highlight the difficulties that mobile agents have to establish a cooperative environment in a spatial setting.

Cooperation is a desirable behavior that is fundamental for the harmonious development of society. However, cooperation may easily fall prey to exploitation by selfish individuals who only care about short-term gain. For cooperation to evolve, specific conditions and mechanisms are required, such as kinship, direct and indirect reciprocity through repeated interactions, or external interventions such as punishment. Reputation in the case of repeated interactions^1^ and assortment mechanisms that favor cooperator-cooperator interactions^2^ are the key, as first shown by W.D. Hamilton^3^ in the case of genetic relatedness. In principle, positive assortment among cooperators might also result when agents interact through network relationships. The reviews^4^,^5^ aptly summarize the vast amount of work that has been accomplished in the last two decades on the study of such cooperation-enhancement mechanism. Theory and numerical simulations suggest that network reciprocity can explain the evolution of cooperation in a population of self-regarding agents under certain circumstances (see, e.g.,^6^,^7^,^8^,^9^). But what can be said about real people? Recent research tested these predictions by means of targeted experiments with humans in the laboratory, in which the subjects were connected in specific network structures, including large-scale ones^10^,^11^,^12^,^13^. Surprisingly, these studies found that neither homogeneous nor heterogeneous network structures promote cooperation to a significant extent^10^,^11^,^13^,^14^,^15^,^16^.

However, when people are allowed to change their neighborhood by deleting unsatisfying relationships and trying to form better ones, theoretical and numerical models agree in concluding that cooperation may evolve (see, e.g.,^17^,^18^,^19^,^20^ among others). Remarkably, and in contrast to the static case, empirical laboratory tests of dynamic settings performed in the last few years did confirm experimentally that fluid networks allow cooperation to evolve^21^,^22^. In other words, unless there is uncertainty about the behavior of neighbors that is costly to resolve^23^, dismissing relationships towards exploiting defectors as a form of direct punishment usually allows cooperation to prevail, even when rewiring an undesired link requires paying a cost^24^. This is certainly an encouraging result and the laboratory settings used, except for their reduced size, do match to a significant extent in spirit present-day internet-mediated relational social networks in which links are essentially independent of location in physical space.

Nevertheless, there also exist many situations in which geographical space does play a fundamental role in biological and ecological systems, as well as human societies. When agents find themselves in geographical space they usually can move around. Many examples can be found in mobility patterns of human populations, e.g.^25^, in engineered systems such as ad hoc networks of mobile communicating devices (see e.g.^26^,^27^), and mobile robot teams^28^,^29^ among others. In the case of agents interacting in physical space through game-theoretical principles, mobility might turn out to have an important effect for cooperation but, surprisingly, there has been comparatively little research in evolutionary game theory on this subject. Early on fixed regular lattices were used to represent spatial interactions in populations in a simplified manner in evolutionary games by Axelrod^30^ and by Nowak and May^31^. They showed that, even when the game is one-shot, i.e. pairs of players interact anonymously, cooperation can evolve thanks to positive assortment among cooperators. A summary of this and other early work on grids is provided in^32^. However, more recent extensive numerical simulation work showed that the gains in the paradigmatic Prisoner’s Dilemma game depend on the players’ strategy update rule used^7^. Mobility of individuals can readily be introduced in these models through the use of a *diluted* grid, i.e. a lattice only partially filled by players such that agents can change location by moving to an empty cell in the grid. Mobility may have positive or negative effects on cooperation, depending on several factors. An early study was carried out by Enquist and Leimar^33^ whose conclusion was that mobility may seriously restrict the evolution of cooperation as a result of randomization and invasion by defectors. In the last decade there have been several new studies of the influence of mobility on the behavior of various games in spatial environments, either in the case in which agents essentially perform random walks, or when they move according to heuristic strategy-driven considerations. Random diffusion of agents playing games has been studied in two-dimensional diluted grids^34^,^35^, as well as in continuous space^36^,^37^. Random diffusion has been thought to hinder cooperation by randomizing the population and reducing the possibility of cooperator cluster formation. However, the works^34^,^35^,^36^ show that cooperation can be maintained with respect to the static case and sometimes even enhanced for low mobility, depending on the strategy update rule used by the agents. When agents are allowed to move according to some simple heuristic rule instead of randomly, it has been shown that the population may evolve a certain degree of self-organization^38^,^39^,^40^,^41^,^42^,^43^,^44^. Clearly, intermediate situations also exist whereby the movement is partly random and partly contingent on some criterion.

A general conclusion about all the above works is that it is far from clear whether cooperation may be helped or hindered by random mobility as the result depends on many factors. Only purposeful contingent movements seem to be able to lead to highly cooperating population states^38^,^39^,^40^,^41^,^42^,^43^,^44^. In view of the practical importance of individuals’ mobility in geographical space it appears that, like the case of static and dynamic networks, some light could be shed on the issue by experimental work with human subjects. Indeed, to our knowledge, no laboratory experiment of this kind has been published to date. In the rest of the paper we discuss our experimental setting with its limitations and constraints, the results we obtain, and some possible interpretations of the observed player’s behavior.

## Experimental Design

Our experimental setup is based on the customary Prisoner’s Dilemma (PD) game^30^,^45^. In this two-person game players must decide whether to cooperate or to defect. If both cooperate, each gets a payoff *R*. If one defects and the other cooperates, the defector gets *T* and the cooperator receives the payoff *S*. If both defect, each gets *P*. Since *T *> *R *> *P *> *S*, defection is a dominating strategy and a rational payoff-maximizing player will choose to defect, although mutual cooperation yields a higher collective payoff, whence the dilemma. Evolutionary reasoning leads to the same result, as defectors will reproduce at a higher rate due to their superior payoff^46^. This simple game is a good metaphor for the tension that exists between socially desirable outcomes and self-interested individual actions. In our experiment, subjects played a series of two-person Prisoner’s Dilemma games with their immediate neighbors in the network. We took *T* = 8, *R* *=* 6, *P* *=* 2, and *S* = 0.

Our experimental setting is new in that we introduce a virtual spatial dimension represented by a square grid that wraps around itself into a torus, as in the experiments reported in^11^,^13^, but we also allow for player movements, as explained below, like in the coordination experiment reported in^47^.

After having given the necessary information to the subjects (see SI sect. 1 for details), the experiment unfolds as follows. At the beginning, each of the twenty participants occupies a randomly assigned cell in an 8 × 8 grid giving a partially filled grid with a density of players ρ = 20/64 = 0.3125, i.e. about 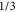. Participants must then choose a cooperate or defect strategy. These are called simply A and B in the experiment so as not to create a psychological bias (the A and B meaning is switched for different groups).

**Figure 1:**
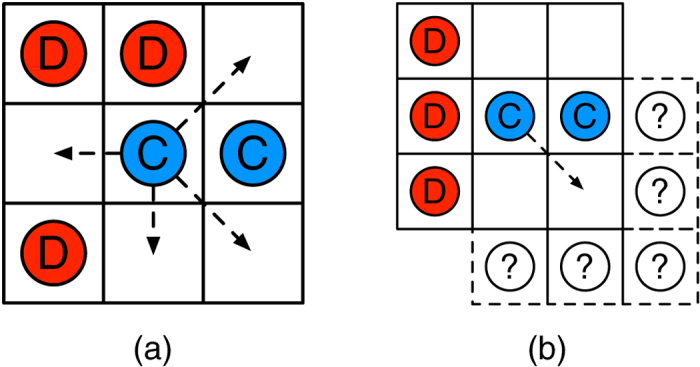
(**a**) An illustration of the neighborhood seen by participants (they don’t see the arrows). In this case, the central player could choose to switch to defection, and/or to migrate to one of the empty cells indicated by the arrows. (**b**) When it comes to decide whether to move or not, if the central player moves to the cell indicated by the arrow, it will keep the other cooperator as a neighbor while the other positions may contain either players with an unknown strategy, or are empty.

Before deciding on the next actions to perform, subjects see the following information on their screen: their current strategy, their cumulated gain in conventional points (which is translated into actual monetary payment at the end of the experiment), and the position and strategy of their current neighbors in the 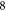-cell neighborhood as schematically shown in Fig. 1a. As in most previous works, each player uses the same strategy against all her neighbors. Using different strategies against different neighbors (see e.g.^48^) might be more adequate if the neighbors were distinguishable, but not in our setting because players do not have labels to tell them apart and, owing to mobility, they can leave their positions and be replaced by other anonymous players.

For each player, a round of the treatment consists first in deciding whether to keep the current strategy or to switch to the other one. Next, each player decides whether or not to move to a neighboring empty cell, if any. To avoid multiple cell occupancy, if more than one player wants to move to the same cell only one is chosen at random. An illustration of a possible move decision by the central 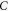 player is given in Fig. 1b. For human players we assume that the decision of the cell to move to might be made according to some heuristic rather than fully randomly. Finding those heuristics and comparing them with theoretical models would be one relevant contribution of our experimental approach. Furthermore, the decision will be done under some uncertainty represented by the “?” symbols in the figure, which stand for a possible new neighbor with an unknown strategy or an empty cell. At the end of this process, participants accumulate their payoffs obtained by playing the PD in pairs against all their non-empty neighbors. These decisions are taken synchronously by all participants. After all players have completed these steps, another round begins.

Our spatial simulated setting is a necessary but, in our opinion, acceptable compromise between the obvious technical limitations of the laboratory and the much more general numerical simulation models that have been proposed in the literature^34^,^35^,^38^,^39^,^40^,^41^,^43^,^44^. Participants in the laboratory setting can only perform jumps to neighboring empty cells like in^34^,^35^,^42^. Given the number of available cells (64), long jumps like those of^41^,^44^ are out of the question, given the available laboratory equipment, for both technical and financial reasons because too many participants would be required.

In the following sections we first present our experimental results and then we discuss the global statistical behavior of the participants. Starting from these results we then try to uncover the rules of the participants’ decision making.

## Results

**Figure 2:**
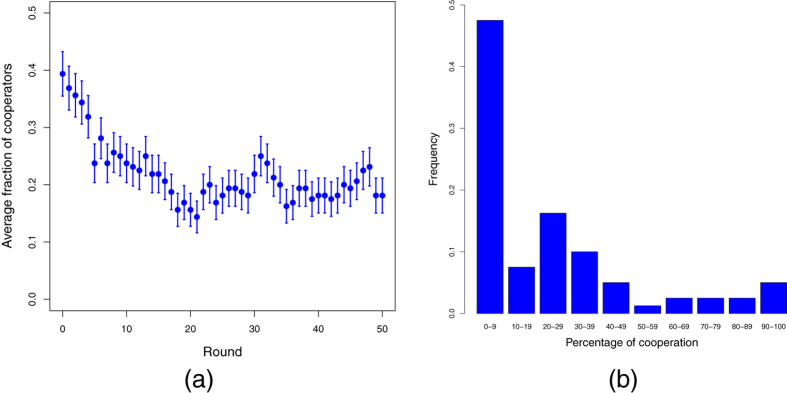
(**a**) Average fraction of cooperators as a function of time for all treatments. (**b**) Overall participants’ frequency of cooperation in deciles cumulated for all treatments. Most participants belong to the 0-10 decile and thus cooperate very little. In the low-middle range (10-50) we find participants who behave as “moody conditional cooperators” (see text).

We now turn to the discussion of the main experimental results. The first and most important observation is the global amount of cooperative acts in the population averaged over all treatments. This is shown in Fig. 2a where it is seen that cooperation could never increase past the initial fraction of about 0.4 and stabilized itself around 0.2 Although it is well-known that, contrary to theoretical predictions, cooperation almost never goes to zero in experimental work on the PD, it appears that migration does not help cooperation to evolve, at least in the present experimental setting. Indeed, after a transient period of a few rounds, typical cooperation levels are similar to those found by Traulsen *et al*.^13^ in their experiment on a fixed full grid in spite of the widely different settings. Related experiments have been performed by Gruji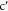 *et al*.^11^ and Gracia-Lázaro *et al*.^10^ in which much larger full grids were used and, as in^13^, participants can be reassigned randomly to a different position but no autonomous mobility in the sense of our setting is provided. Here again the fraction of cooperation tends to stabilize around 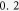 after a transient period.

Another view of the global cooperation results is given in Fig. 2b where we report the fraction of participants as a function of the percentage of cooperative actions cumulated over all participants and all sessions. For instance, the highest bar to the left represents the fraction of participants that cooperated between 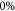 and 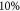 of the times. Although it is clear that defection prevailed among the participants, it is interesting to note the presence of a region comprised between about 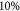 and 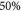 where people cooperated a fair amount of times. Another remarkable thing is that there exists a small fraction of players that cooperate more than 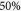 and even some participants that cooperate nearly always, as shown by the last bar to the right.

**Figure 3:**
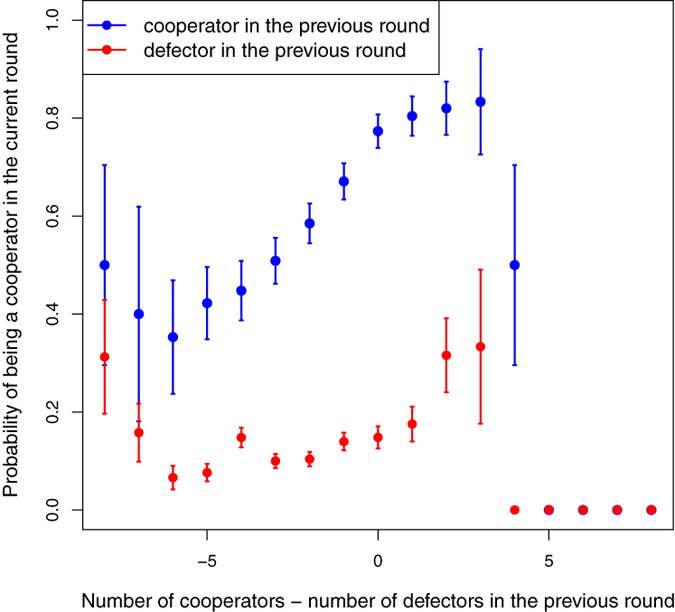
Probability of cooperating in the current round having cooperated (blue curve) or defected (red curve) in the previous one, as a function of the difference between the number of cooperators and defectors in the neighborhood in the previous round. The data are those of the participants cooperating between 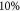 and 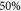 of the time (see Fig. 2b).

We have looked in more detail at the behavior of the group cooperating between 10% and 50% of the time in order to compare it with the findings of earlier experiments on the PD^10^,^11^,^14^,^15^,^16^. As Fig. 3 shows, individuals in this group show a behavior consistent with the moody conditional cooperation found in those previous experiments: a cooperative decision is followed by another one with relatively high probability, the higher the number of cooperative partners in the previous round. On the contrary, a defection is most likely to be followed by another one. While we here show the dependence in terms of the difference between cooperators and defectors in the neighborhood, plotting the same two quantities as a function of the number of cooperative neighbors yields qualitatively the same results (see SI, Fig. S3). Therefore, we conclude that, even if subjects can move around in our spatial setting, the decision to cooperate or to defect is very much determined by a reaction to the observed behavior in the neighborhood and the players’ own mood, as in the PD experiments on fixed lattices.

**Figure 4:**
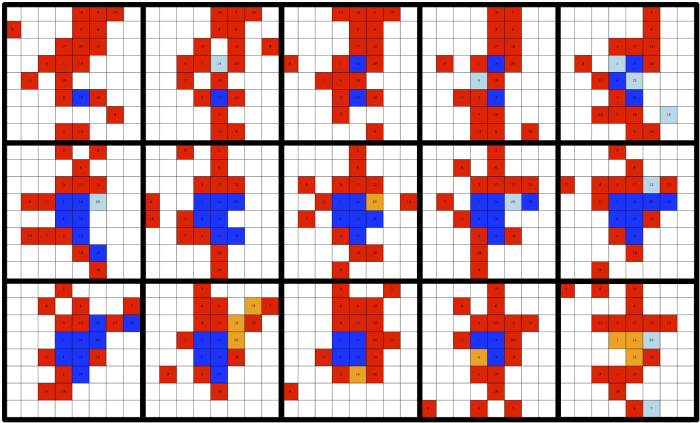
Snapshots for one of the treatments going from round 25 (upper left) to round 39 (lower right; time advances from left to right and from top to bottom). One can see the beginning of the formation and the subsequent dissolution of a cooperator cluster. Cooperators are in blue and defectors are painted red. A light blue cell stands for a cooperator that was a defector in the previous round and an orange cell indicates a defector that was a cooperator in the previous round.

The evolutionary process is a complex one but we hope to offer a gist of it with the time evolution shown in Fig. 4. This figure represents a particular instance in our experiment but it is absolutely typical of all treatments. The time frames in the figure represent rounds 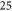 to 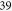 of the first treatment. As can be seen from the sequence of snapshots, sometimes a few subjects that happen to be close to each other cooperate simultaneously and initiate a cluster of cooperators. However, contrary to the pioneering suggestion in^31^, these clusters do not spread: they are not stable and, after a few rounds they vanish as cooperators become tired of being exploited by defectors at the boundary. This phenomenon, which we insist we have observed in most sessions (see SI, sect. 3 for more snapshots), shows the stability of the asymptotic state of the population or, in other words, that once a defective behavior pattern sets in, it is practically impossible to revert it to a cooperative one.

Having discussed the emergence and decay of cooperation in general, taking into account previous experiments and intuitions, we now turn to the unique feature of our setting: the possibility of changing position that is offered to the participants and its possible effects on cooperation. In all our sessions, we have observed that players are rather mobile during the first rounds but, as time goes by, mobility decreases slightly and they tend to settle at some position in space, although movement never ceases until the end of the experiment (see SI, Fig. S4). In fact, a cooperator, unless she finds herself in a very favorable cooperation environment, tends to escape from incoming defectors. Conversely, a defector moves because she is always seeking a cooperation environment in order to accumulate more payoff. We also note that mobility and the incentive to aggregate in order to get a larger payoff cause the mean degree to increase from the initial value of 2.41 to about 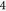 (see SI, Fig. S5).

**Figure 5:**
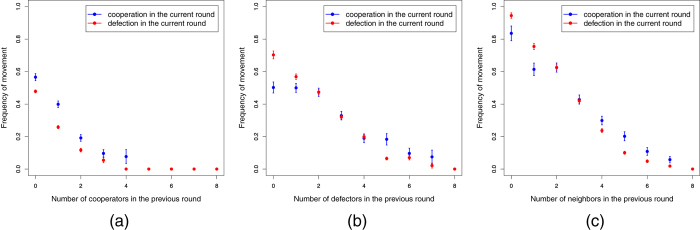
Average mobility of players in their neighborhood. Frequency of movement of cooperators and defectors as a function of (**a**) the number of cooperators in the previous round, (**b**) the number of defectors in the previous round, and (**c**) the total number of neighbors in the previous round.

Besides the global figures, it is perhaps of more interest to plot the average mobility behavior of cooperators, respectively defectors, as a function of the composition of the local neighborhood they experience, since this is the only information on which they can base their decisions (let us recall here that we do not show information on the earnings of their partners; in this respect, it is important to keep in mind that it has been recently shown that players do not take their partner’s payoff into account when making their choices^15^). Figure 5 shows the corresponding plots for the number of cooperators (a) and defectors (b) in the neighborhood, and the total number of neighbors (c), in the previous round.

In Fig. 5a, we observe that the mobility of both cooperators and defectors is lower on average than in Fig. 5b and decreases faster with the number of cooperating neighbors. This is in agreement with our interpretation that both cooperators and defectors aim at a cooperator environment as far as possible. Cooperators move more than defectors because the latter are more satisfied with a given number of cooperator neighbors. Figure 5b is somewhat more difficult to understand, but it shows that the mobility behavior of both cooperators and defectors is similar when the number of defecting neighbors is between two and four; however, the mobility of cooperators increases when this number is more than four. In this case cooperators feel exploited and try to evade defectors although the average number of free cells around them tends to decrease. In the other limit, i.e., when there are only a few defectors around, cooperators are more patient in tolerating exploitation while defectors move more frequently, searching for cooperators to exploit. This happens because with a mean degree tending to about four, there are about one or two other cooperators around the focal one and so the latter is relatively satisfied and shows less tendency to move. A caveat is in order here regarding mobility when many neighboring sites are occupied, irrespective of the actions of the individuals occupying them: in those situations, players have less options to move (in fact, if they were completely surrounded, which is an uncommon phenomenon, they would not be able to move at all). This is reported in Fig. 5c where mobility of players of the two kinds becomes more and more hindered as the number of neighbors grows and, correspondingly, the number of free sites becomes small. This is an extra factor influencing their behavior and can also be responsible for part of the decay of the mobility in panels (a) and (b).

**Figure 6:**
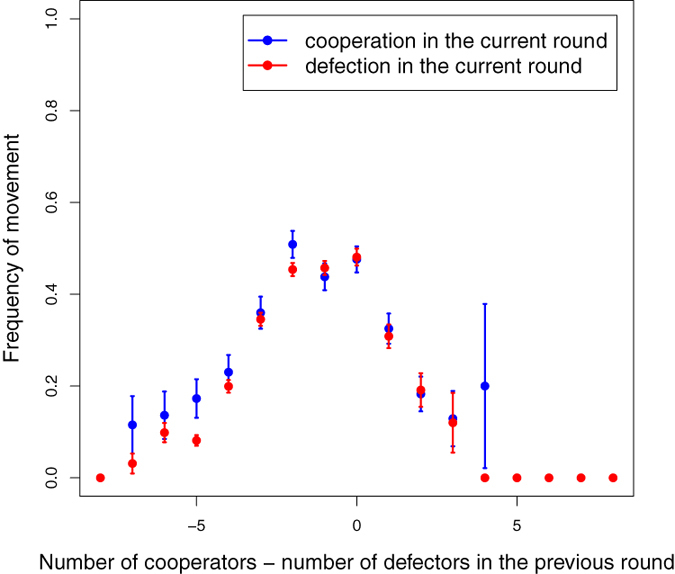
Frequency of movement of cooperators and defectors as a function of the difference between the number of cooperators and defectors in their neighborhood in the previous round.

A complementary view of local mobility is provided by Fig. 6, in which the average mobility of both cooperators and defectors is reported as a function of the difference between the number of cooperators and defectors in the neighborhood of the focal player. It appears that mobility is maximal when this difference is around 0, meaning one cooperator less than the number of defecting neighbors. This is understandable because in this situation neither a cooperator nor a defector is satisfied. For instance, if a cooperator has a defector and another cooperator in his neighborhood, he will tend to move closer to the cooperator. However, as discussed above, when there are many cooperators and defectors in the neighborhood, most of the time the movement cannot take place, either because of collisions or because of lack of free cells. At the extremes of the curve, where the difference is large in absolute value, either the neighborhood is too crowded to allow movements, or the cases are rare and have a high standard deviation, or they have not been observed. An interesting observation arising from Fig. 6 is that cooperators and defectors exhibit the same mobility, within the error bars, in the whole range of differences, which means that even if they are likely to move for different motives, their behavior could be described by the same, simple heuristics, opening the door to simulating larger systems and studying other parameter values.

## Discussion

In this paper, we have presented the results of an experiment intended to shed light on the hitherto unclarified issue of the relevance of mobility in a geographical context to cooperation. In particular, important differences between random and purposeful motion in their ability to support cooperative interactions had been reported from a theoretical viewpoint, but experimental counterparts to those results were lacking. In the context of this previous literature, our most relevant result is that mobility does not promote cooperation: in fact, as in most experiments involving a Prisoner’s Dilemma, we have found that the fraction of cooperators decays from an initial value close to half the population to residual ones of approximately a 20%, a value that is almost universally found in the laboratory. In fact, a vast majority of players can be classified as defectors or as moody conditionally cooperators, i.e., as players whose probability of cooperation depend not only on the actions of their partners but on their own previous actions, a type first identified in^10^,^11^,^15^. This indicates that the possibility to move around in space does not change very much the way players choose their actions.

Remarkably, our experiments also contribute to the understanding of the possible assortment of cooperators in order to support cooperation. The numerical simulations reported in^31^ suggested that cooperators may survive by forming clusters in which they mainly interact with other cooperators. In our experiments, we have indeed observed that such clusters appear with non-negligible frequency; however, their lifetime is quite limited because the possibility to move allows cooperating agents at the boundary of the cluster to separate from it to severe their interactions with defectors, or to choose defection themselves. A dynamical network view can also be taken considering that, at each discrete time step, one could draw a spatial graph in which edges connect players that are neighbors. At the next time step, owing to mobility, some links might disappear while new ones may be formed. This, except for the topological constraints imposed by two-dimensional space, compares with dynamical relational networks. In contrast to what has been observed on the latter^21^,^22^, where allowing players to cut and make links at will does lead to clustering of cooperators and to an increase of the cooperation level, in our experiments we measure a much lower amount of cooperation. The reason can be traced back to the fact that, if links evolve indirectly by motion of the players in geographical space, they cannot be cut one by one, and when moving away from defectors cooperators also cut their links to cooperators. Therefore, we conclude that for clusters to be an important factor in the promotion of cooperation, individuals must have complete control on their choices of partners, a condition that has never been put forward before.

Regarding mobility, we have found that players move considerably at the beginning of the experiment, but the average fraction of individuals deciding to move decreases and by the end of the experiment only some 10%-20% of players are moving. We believe that this behavior is connected to the observation in the previous paragraph: players realize that the decision to move has very frequently pros and cons as it affects their connections in an indiscriminate manner, and at some point they conclude that they are not going to find a safe haven against defectors. On the other hand, it is worth noticing that in our experiment there is no punishment for interacting with a defector, and therefore all the residual motions observed in late stages must arise from spite, i.e., from subjects preventing others to benefit from them even if they are not harmed by those partners’ actions. This is in agreement with our finding that cooperators tend to move somewhat more often that defectors, implying that while the latter just move trying to find others to exploit, cooperators have the additional motivation to punish defecting partners. In addition, we have also observed that mobility of all players is maximum when there is more or less the same number of cooperators or defectors in the neighborhood. Of course, to interpret these results one needs to bear in mind that when a player has many partners her mobility is also reduced by the lack of available cells to move to. With this caveat, it appears that when the number of cooperators and defectors is approximately the same around a given subject, she will try to move to increase her interaction with cooperators irrespective of her own action, as can be expected. On the contrary, when there are many neighbors of the same type, mobility becomes less relevant and perhaps impossible, this being the reason why we observe a maximum.

In conclusion, we stress that the interaction between behaviour and mobility does not seem to increase the level of cooperation in a human population set on a geographical framework. The main reason for this phenomenon turns out to be the fact that setting and breaking links cannot be done independently for every player as the mechanism for rewiring is motion in space. Interestingly, these results pose important questions about the emergence of cooperation in neighboring human groups, which could be most relevant in interactions in a socio-ecological context among hunter-gatherer groups, either in our recent evolutionary past or in presently existing populations. Furthermore, the type of mechanism we have unveiled with our experiment is most certainly not a very sophisticated way to make decisions, and therefore similar conclusions might apply to spatially structured populations of many other animals or bacteria. In this respect, our findings may provide a new perspective to interpret observational data on cooperative behavior in social animals, pointing to other behavioral traits (e.g., in terms of deciding to move or to change action) that coevolve with the geographical distribution of the population in its ecological environment.

## Methods

The use of human subjects in this experiment was approved by the Ethics Committee of the University of Lausanne and it is in accordance with all relevant guidelines and regulations. We conducted a total of four experimental sessions in February-March 2014 in a specially equipped laboratory using the z-Tree environment^49^. The participants were fully informed of the nature of the experiment and signed an informed consent to participate. Their anonymity was guaranteed at all stages of the experiment. Each session involved 20 participants and a total of 80 subjects took part in the experiment. Each session consisted of two repetitions of the same treatment played by the same group but with new random initial conditions. An experimental treatment consists of 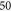 rounds but, to avoid end-of-treatment effects, this number is unknown to participants who are told that there will be between 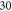 and 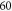 rounds. Participants were recruited using ORSEE^50^ from a subject pool that includes students from several faculties of the University of Lausanne and of the Swiss Federal Institute of Technology (EPFL).

Students read a detailed description of the experiment before playing the game. After reading the instructions, subjects had to respond to a set of control questions that insured common understanding of the game and the computation of payoffs. An English translation of the instructions distributed to subjects is provided (see SI sect. 1). Each session lasted about 60 minutes. Participants earned a certain number of points during the experiment and their final score in points was converted at an exchange rate of 1.- CHF = 50 points. The average gain per student was 22.5 CHF (about 19 EUR).

## Author Contributions

A.A., A.S., and M.T. conceived and designed the experimental setting. A.A. prepared and performed the experiments. A.A., A.S., and M.T. analyzed the data and wrote the paper.

## Additional Information

**How to cite this article**: Antonioni, A. *et al*. Short-Range Mobility and the Evolution of Cooperation: An Experimental Study. *Sci. Rep.* **5**, 10282; doi: 10.1038/srep10282 (2015).

## Supplementary Material

Supplementary Information
