# Supplementary material for: Short-Range Mobility and the Evolution of Cooperation: An Experimental Study
Source: Sci Rep. 2015 May 20;5:10282. doi: 10.1038/srep10282 (PMC4438713; doi:10.1038/srep10282)
Supplement: Supplementary Information [file srep10282-s1.pdf]

Short-range mobility and the evolution of cooperation: an experimental study
Supplementary Information

A. Antonioni, A. Sánchez, and M. Tomassini

February 15, 2015

The supplementary information (SI) provides details on the exact instructions form that participants received (section 1), complementary results of experimental data not shown in the main text (section 2), and an overview of some typical configurations observed during the experiment (section 3).

1 Instructions form

Each participant read the following set of instructions in detail before the experiment started. Here we present the instructions form for the global treatment case. The only difference between the two treatments is that in the local treatment case participants never know the current number of conventions present in the population at each round.

(The following instructions were originally written in French.)

Explanation for the first part of the experiment

Welcome to this experiment!

You will have to make decisions that will affect your income as well as the income of other participants. Although we express all income in terms of points, these points will be exchanged at the end of the experiment using the following exchange rate:

50 pts. = 1.- CHF

From now on, it is **strictly forbidden to talk with other participants**. If you have any questions, please contact the assistants. If you do not follow this rule, we will have to exclude you from the experiment.

In this study, each one of the 20 participants is placed on a cell of a chessboard, i.e. a 8×8 grid (see below).

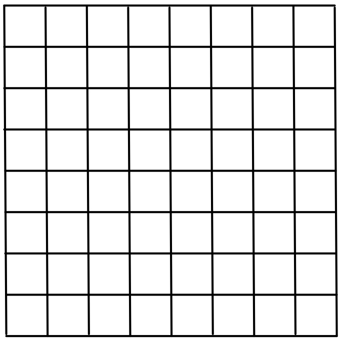

Each cell cannot be occupied by more than one participant. Consequently, 64 (total number of cells) $-$ 20 (participants) = 44 cells will always be empty.

During each round of this experiment, you interact with participants placed in a cell of your neighborhood. Your neighborhood is defined as the 8 cells of the chessboard that are around your current position. You can find a representation of your neighborhood here below: your position is in the middle (X) and 8 cells (x) are around you.

x	x	x
x	X	x
x	x	x

<u>**Border conditions**</u>: if your cell is one at the border of the grid or one of the four corners, your neighborhood always contains 8 cells since the grid is virtually wrapped around itself as in the figures here below.

			x	x	x		
			x	x	x		
			x	X	x		

x						x	X
x						x	x
x						x	x

However, during the whole experiment, you will only see your neighborhood and you will not be able to see what happens in the rest of the chessboard.

What is it about?

<u>There will be a number of rounds between 30 and 60</u>. In the first round, you must choose a profile between options **A** and **B** but, as you will see below, this may change during the experiment.

Your gain for each round depends on the combination of your profile and those of your neighbors. Here below we explain the gain you and one of your neighbors get for each possible combination of your profiles:

- Your profile is **A**, the profile of your neighbor is **A**: you gain **6** points and your neighbor also gets **6** points.
- Your profile is **A**, the profile of your neighbor is **B**: you gain **0** points and your neighbor gets **8** points.
- Your profile is **B**, the profile of your neighbor is **A**: you gain **8** points and your neighbor gets **0** points.
- Your profile is **B**, the profile of your neighbor is **B**: you gain **2** points and your neighbor also gets **2** points.

The following examples illustrate your gain in a round. Your profile is shown in the central cell represented by a capital letter, while your neighbors' profiles are represented with lower case letters.

Example 1: Your profile is **A** and you are in the following situation:

	a	a
	A	
		a

Your gain for this round is: $6 + 6 + 6 = 18$ points.

Example 2: Your profile is **A** and you are in the following situation:

	b	
b	A	
b		

Your gain for this round is: $0 + 0 + 0 = 0$ points.

Example 3: Your profile is **B** and you are in the following situation:

		a
a	B	
a		

Your gain for this round is: $8 + 8 + 8 = 24$ points.

Example 4: Your profile is **B** and you are in the following situation:

	B	b
b		b

Your gain for this round is: $2 + 2 + 2 = 6$ points.

Example 5: Your profile is **A** and you are in the following situation:

	a	b
a	A	b
a		

Your gain for this round is: $6 + 6 + 6 + 0 + 0 = 18$ points.

Example 6: Your profile is **B** and you are in the following situation:

a		b
b	B	b
		a

Your gain for this round is: $8 + 8 + 2 + 2 + 2 = 22$ points.

Example 7: Your profile is **A** and you are in the following situation:

	A	

Your gain for this round is: 0 point.

Example 8: Your profile is **B** and you are in the following situation:

	B	

Your gain for this round is: 0 point.

What will you do ?

At each round, you take two decisions:

Profile switching

You may decide to change your profile switching to the other profile. This profile will be yours until you decide to change again.

Movement

You may decide to move yourself to an empty cell in your neighborhood, if any. If you try to move to a cell which is already occupied by another participant, your displacement won't be accepted. Moreover, if more than one participant want to move into the same cell, only one participant will be chosen while the others won't move for that round.

You will take these decisions using the following screen (Fig. S1):

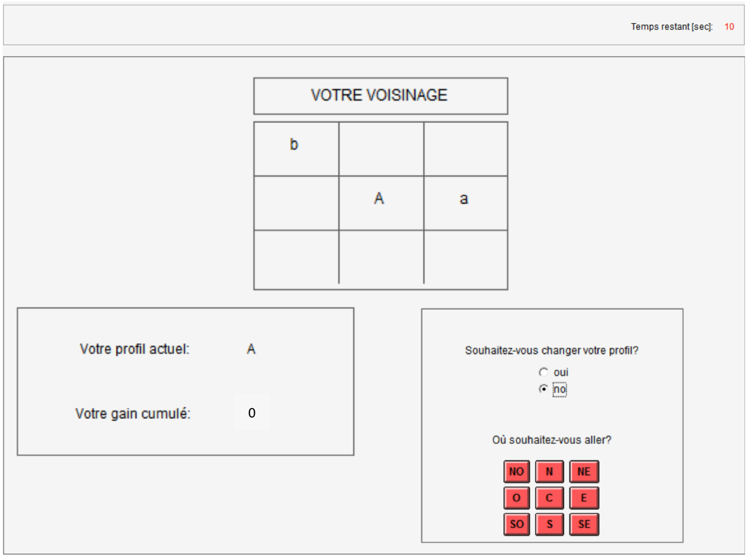

Figure S1: Your neighborhood is shown at the top of the screen, while your current profile and your cumulated payoff are illustrated on the left of the screen. On the right side you can choose to change your profile and if you want to move during this round. Note that if you want to keep your position for the current round you may click on the "C" button.

At the end of each round, you will see your final profit for that round and you will be informed about your new neighborhood. This information will be shown you as follows (Fig. S2):

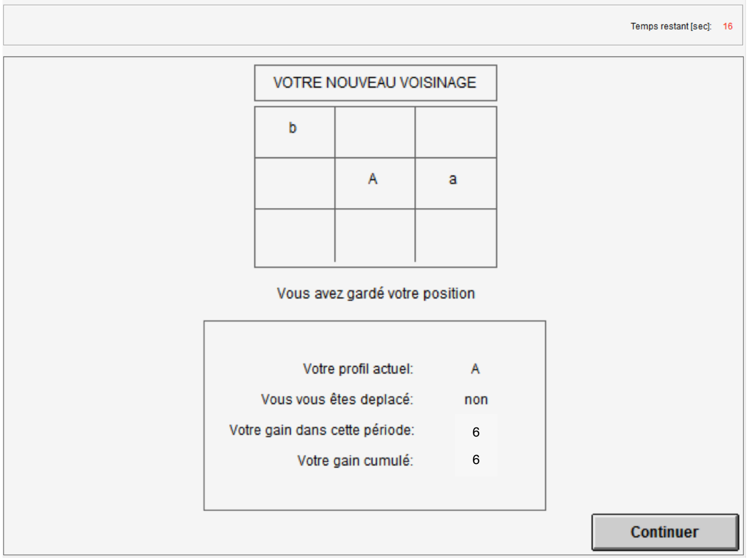

Figure S2: Your new neighborhood is shown at the top of the screen. Your current profile, a message for your eventual displacement, your profit for the current round and your cumulated payoff are shown at the bottom of the screen.

Did you correctly understand the instructions?

Before starting the experiment, we would like to be sure that you and the other participants have correctly understood the decisions that you are going to make. To this end, please answer the questions that will appear on the screen.

(Then, all the participants must answer correctly to 7 trial questions before the first treatment begins. After have played for 50 rounds, participants receive another instructions form (here below) before playing for another treatment. Each group of 20 participants play for a total of 2 treatments.)

Explanation for the second part of the experiment

This part of the experiment is almost the same of the previous one. At the beginning, you must decide your starting profile and your position on the chessboard will be randomly initialized as before.

2 Complementary results

Here we show the complementary results of the experimental study.

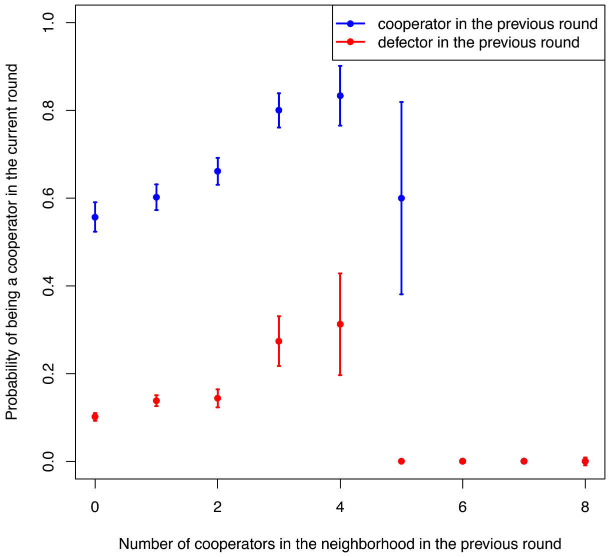

Figure S3: Probability of cooperating in the current round having cooperated (blue curve) or defected (red curve) in the previous one, as a function of the number of cooperators in the neighborhood. The data are those of the participants cooperating between 10% and 50% of the time.

3 Typical snapshots

This section presents two complete treatments of 50 rounds we observed. We have run 8 treatments. Each group of 20 participants played the same setting for two times.

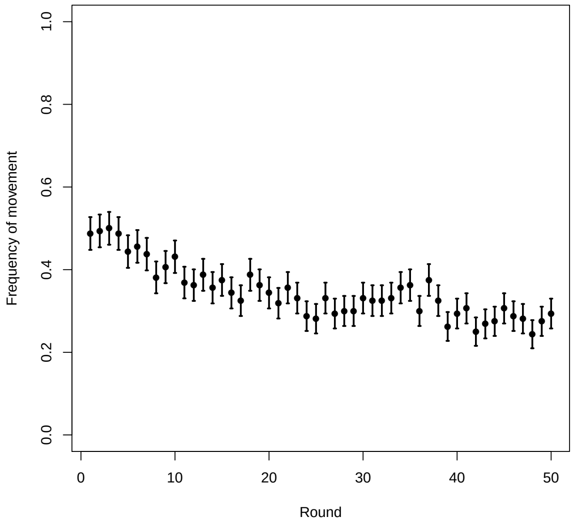

Figure S4: Average mobility of participants per round during the experiment cumulated over all treatments.

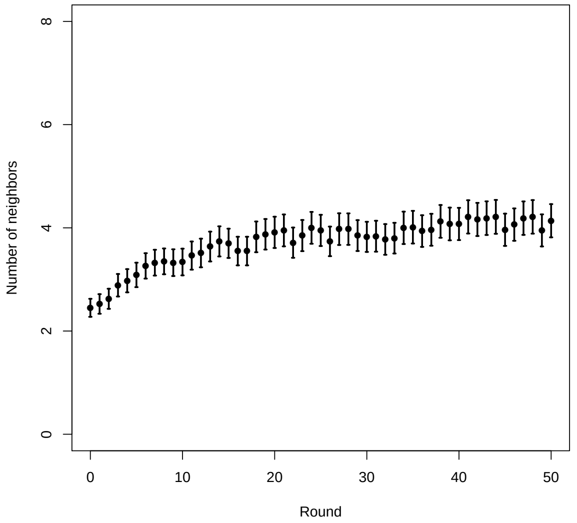

Figure S5: Evolution of the average number of neighbors (mean degree) cumulated over all treatments.

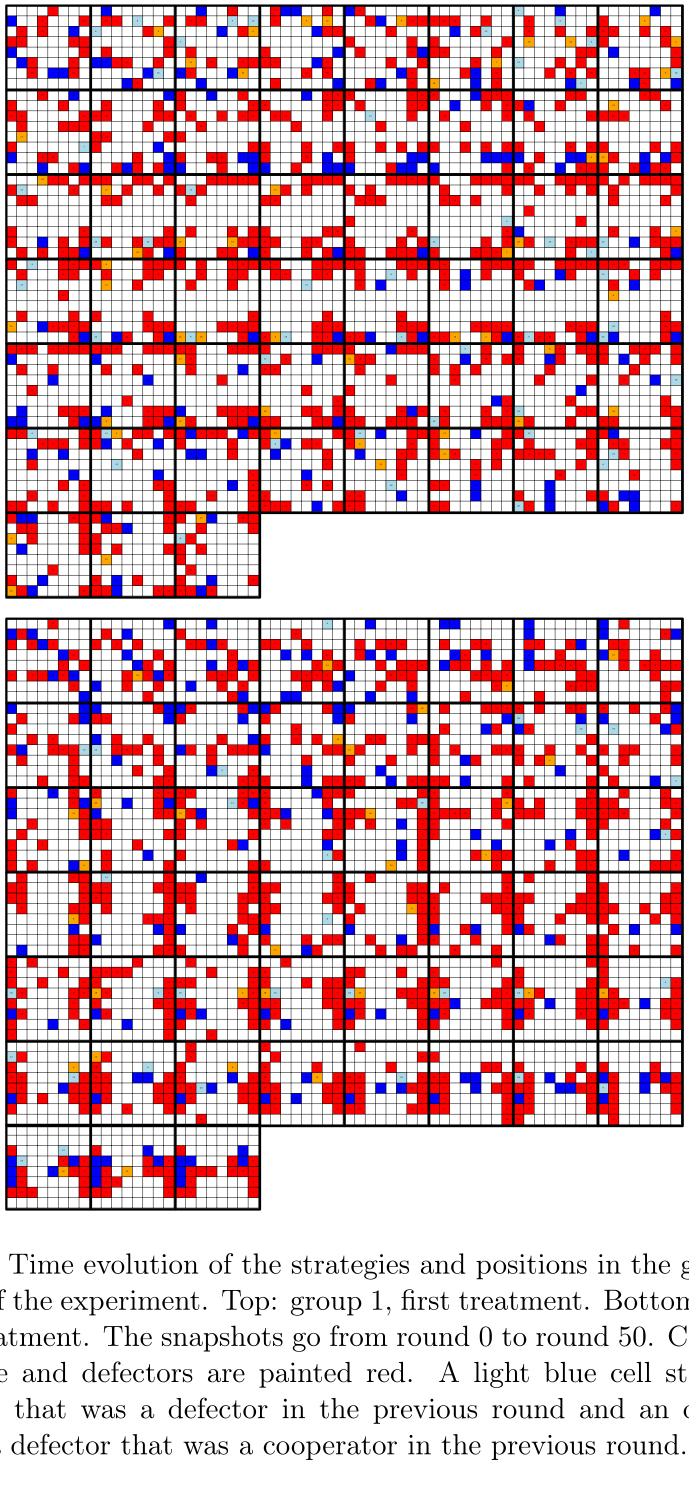

Figure S6: Time evolution of the strategies and positions in the grid during a session of the experiment. Top: group 1, first treatment. Bottom: group 1, second treatment. The snapshots go from round 0 to round 50. Cooperators are in blue and defectors are painted red. A light blue cell stands for a cooperator that was a defector in the previous round and an orange cell indicates a defector that was a cooperator in the previous round.
